# New Remedies

**Published:** 1824-06-01

**Authors:** 


### NEW REMEDIES.

1. *Formulary for the Preparation and Mode of employing several new Remedies, &c.* Translated from the French of M. Magendie, by T. C. Haden, with an Introduction and Notes. Small octavo, pp. 110. London, 1823.
2. *On the comparative Virtues of different Kinds of Sarsaparilla. To which is added, an Appendix on Cinchonine, Quinine, and other new Vegetable Principles.* By Mr. John Pope, Chemist. Octavo, pp. 21. London, 1824.

Although we are not so sanguine in our expectations respecting new remedies as either Dr. Magendie or his translator, Mr. Haden, yet we are very far from discountenancing the pursuit of novelty in our therapeutical agents, convinced, as we are, that new and powerful remedies will continue to be elicited in every age to the end of time. The field for discovery is boundless—the materials inexhaustible—the inducements preponderating.

---

"I intend to lay the history of these cases before the public at some future opportunity."

Under such circumstances, and with the aid of a rapidly improving chemical science, what may not be expected from the restless activity of man? There is no good, it is true, without alloy. The same process which evolves the active principles of vegetables, swells the long list of poisons, by which the assassin destroys his victim and the suicide himself! Thus, of all the poisons which the ingenuity or the villainy of man has ever invented, what is so rapidly destructive of human life as prussic acid? In a proper dose, death and deglutition are simultaneous —and the suicide rids himself of existence without a pang, without a consciousness of the awful transition from time to eternity. We have no doubt that this substance will, ere long, be almost the only instrument of *self-destruction*. We have reason to believe that it has been used, most effectually, for this purpose already by a medical man in this metropolis. The late judicial proceedings in Paris, on Dr. Castaing's case, shew that some of the new remedies have been applied to the horrible purpose of murder. But, perhaps, of all substances the strychnine will prove the most dangerous in the hands of the assassin, since the diffusible odour of prussic acid will almost always confine it to the hands of the suicide. The *sixteenth part of a grain* of strychnine will kill a small dog! What a tremendous agent in medicine or toxicology!

Mr. Haden, in his translation, adverts to the objection which has been made to the principle of isolating and concentrating the active parts of medicines, as rendering them less efficacious than when presented to us by the hand of Nature. We perfectly agree with our author, that these active principles of medicinal substances should be looked upon as *simples*, the properties and doses of which are to be ascertained by experience, in the same way as was done with all other remedies. In this point of view, we are likely to derive great advantage from the use of remedies concentrated, and freed from their nauseous and often jarring principles. In many diseases, climates, and individuals, the stomach will not bear medicines of bulk, as the cinchona for instance, and in such cases the new remedies will be of the greatest importance. And even granting that the original properties of drugs should be entirely altered by these new analyses, still we are gainers by all the new properties developed, as we have, after all, the choice of both forms. In respect to bark, it is now clearly ascertained that the quinine and cinchonine are of great importance in medicine, and we have no doubt but that they will be advantageously employed in numerous diseases where the bark in substance would be totally inadmissible.

The expense of preparing the new medicines has been urged as an objection; but a few shillings are of very little consequence compared with life or health. And we have no doubt that the processes will yet be much simplified so as to enable the operative chemist to furnish the practitioner with the said remedies at a much lower rate than at present.

In the following notice of the publications now before us, we shall enter very little into the chemical or pharmaceutical processes employed in the preparation of the new remedies, but dwell principally on their physiological or medicinal effects. Few of our readers will venture to prepare the remedies in question, but many of them will wish to employ them in practice, when procured from the operative chemist.

I. *Resin of the Nux Vomica.*—So far back as 1809, M. Magendie ascertained that an entire class of vegetables (the bitter strichnos) has the singular property of powerfully exciting the spinal marrow, without involving, except indirectly, the functions of the brain. The alcoholic extract of the nux vomica has since been frequently employed in both partial and general paralysis, but the actual degree of success has not yet been ascertained. The preparation is easily made by exhausting, by repeated macerations, a determinate quantity of rasped nux vomica, in alcohol, and evaporating it slowly to the consistence of an extract. The dry extract may be made by dissolving in water the alcoholic, and evaporating to dryness. The dose of the resinous extract is, from half a grain to three grains in pills. It possesses the same properties as the strychnine, but is much more manageable.

II. *Strychnine.*—In the nux vomica, the bean of St. Ignatius, and the celebrated poison of Java, there is a peculiar vegetable alkali termed strychnine, one of the most virulent poisons in nature, as we have before said. It is soluble in water slightly acidulated, and so intensely bitter, that it is decidedly perceptible when diluted in *sixty thousand* times its own weight of water. We tasted the strychnine itself, as prepared by Mr. Pope, and felt unwell after merely applying a particle of the salt to the tip of the tongue. "I have witnessed," says Mr. Pope, "the sixteenth part of a grain paralyze small dogs in four or five minutes, and kill them in less than half an hour." Its action is peculiar and uniform, paralyzing invariably the hinder extremities.

III. *Morphine and Salts of Morphine.*—In morphine, we have exclusively the anodyne or sedative property of opium. It is obtained by a troublesome process in small regular crystals, but little soluble in water, except with a slight excess of acid.

"The acetate of Morphine," says Mr. Pope, "is the combination of this substance, most universally approved;* it is a very deliquescent salt, and may be exhibited in any aqueous fluid, in doses of from one-fourth of a grain to one grain, or formed into a pill with liquorice powder, treacle, &c. I have adopted a very simple method for separating these principles of opium for pharmaceutical purposes, by treating it repeatedly with sulphuric æther, and subsequently forming it, by solution in distilled water and evaporation in vacuo, to the consistence of an extract. This preparation, which I distinguish as '*Extract of Morphia*,'† may be uniformly substituted for the ordinary *extractum opii*, and the dose of it considerably augmented, without producing nausea, restlessness, &c." 18.

The acetate of morphine is prepared by Magendie, by combining directly, in an evaporating dish, acetic acid and morphine, letting the mixture slowly evaporate to dryness.

*Narcotine* contains solely the stimulating property of opium. In its pure state, a few grains of it are destructive of animal life. It has not yet been employed in medicine.

IV. *Emetine.*—Pelletier and Magendie, about six years ago, ascertained that the different species of ipecacuan owed their emetic properties to a particular immediate principle, which they denominated *emetine.* This substance is much more active than the root itself—is devoid of disagreeable taste, or nauseous odour—and may, they think, be advantageously substituted for ipecacuan on all occasions. The *pure emetine* is a new vegetable alkali, white, pulverisable, unaltered by the air, scarcely soluble in water, but easily so in ether or alcohol. Its taste is slightly bitter—it is soluble in all the acids, resembling veratrine by forming with them a crystallizable combination. Its action is similar to that of the impure, or coloured emetine, but much stronger—two grains of it being sufficient to destroy a large dog. One sixteenth of a grain of it produced vomiting in a man of 85 years of age. To operate in this manner, one grain, or rather less, dissolved in acetic or sulphuric acid, may be given in a draught, in divided doses till the effect is produced.

---

* "The liquor opii sedativus is chiefly an aqueous solution of acetate of morphine in combination with a portion of extraneous coloring matter, and a little alcohol." This is conjecture. For proof, see Mr. Battley's paper in the *Extra Limites* of this Number.—*Ed.*

† "Extractum opii sine narcotinâ would be more correct; but the other is more convenient in prescription."

V. *Alkalis of Cinchona.*—Pelletier and Caventou discovered an alkaline substance in bark, which they denominated *cinchonine.* This they obtained by operating on the grey cinchona (cinchona condaminea.) The yellow bark (cordifolia) furnished an alkali which differed considerably from the cinchonine, and to which they gave the name of *quinine.* Next followed the analysis of the red bark, (c. oblongifolia) which furnished cinchonine in triple the quantity yielded by the grey bark, and double the quantity of the quinine furnished by the yellow bark. Further experiments, on a large scale, have shewn that quinine and cinchonine exist simultaneously; but the cinchonine is, relatively to the quinine, in greater quantity in the grey bark; whilst, in the yellow bark, the quinine so predominates, that the presence of the cinchonine might readily escape notice. We shall give the most recent formula for obtaining both these celebrated salts.

"Boil the bark in alcohol until it loses all its bitterness; evaporate to dryness in a water bath; dissolve the alcoholic extract entirely in boiling water, strongly acidulated with hydrochloric acid; add an excess of calcined magnesia, which, after boiling some minutes, will fix all the red colouring matter, and make the liquid clear. When cold, filtrate and wash the magnesian precipitate with cold water; dry it on a stove; separate all the bitterness by repeated digestions in boiling alcohol; mix the alcoholic liquors, and the cinchonine will crystallize as the fluid cools. The cinchonine, which is thus obtained, still contains a green fatty matter, which may be separated by solution in a very weak acid. If the acid be too strong, it will dissolve a part of the fatty matter, and the intended object will be thus defeated.

"Quinine may be obtained from the yellow bark by a similar process to the one described above." 45.

Cinchonine is white, translucent, crystallizable in needles, and soluble in 700 parts of cold water. If dissolved in alcohol, or rather in an acid, it tastes powerfully bitter, and exactly resembles that of the pale bark. The sulphate and acetate of cinchonine are used in medicine. The first is very soluble in water—the second is much less so, but an excess of acid dissolves it with tolerable facility.

Quinine is white and uncrystallizable—it is as little soluble in water as the cinchonine, and is much more bitter to the taste. It is very soluble in ether, while cinchonine is very little so.

*Sulphate of Quinine.*—M. Henry, jun. has lately made known an expeditious and cheap process for directly obtaining the sulphate of quinine.

"He digests, repeatedly, in hot water, acidulated by sulphuric acid (6 or 8 grammes [gr. 92.66 or gr. 123.55 T.] to each kilogramme [oz. 32.17 T.] of distilled water). He blanches the liquors by means of hot lime, and washes the precipitate to separate the excess of lime. He repeatedly digests this precipitate, when well drained, in alcohol at 36° (.837). He then obtains, by distillation, a brown viscid matter, which becomes brittle when cold, and is very bitter. He digests it in hot water, acidulated by sulphuric acid, and the liquor, when cold, gives perfect crystals of pure sulphate of quinine. He has not succeeded so well in extracting the sulphate of cinchonine from the grey bark by this mode of preparation.

"The sulphate of quinine obtained in this way, is in the form of white crystals, which are entirely soluble in water; little so, however, in cold water, but more so in boiling, and especially in weakly acidulated water.*" 48.

*Acetate of Quinine.*—The characteristic of this salt is the great facility with which it crystallizes. It is little soluble in cold, even with an excess of acid.

*Physiological Action of the Salts of Bark.*—A considerable number of experiments have now been made to determine the medical or physiological effects of these medicines, and it is well ascertained, that they possess the properties of the cinchonas, and, consequently, that they may, in almost all cases, be substituted for them. It is, in the first place, desirable to ascertain the precise dose of all active remedies, and this is done better with the alkalis in question, than with the bark from which they are obtained, and in which they exist in different quantities according to the state of the bark. In the second place, it is often, we might say always, desirable to administer medicines in a small volume and agreeable form. In these respects the salts possess great advantages. Chemistry, therefore, has done much service to medicine, by separating the active principles of vegetables from the inert substances with which they are combined in a natural state.

The sulphates of quinine and cinchonine may be given in doses varying from one to ten grains in the 24 hours. Some

---

* "Dr. Paris gives the above process with some variation. He directs two pounds of the powdered bark to be boiled for half an hour in sixteen pints of distilled water, acidulated with two fluid ounces of sulphuric acid. The quantity of lime recommended is half a pound, or enough to render the solution of a dark brown, and to produce a reddish brown precipitate. He says that the two pounds yield ʒv. or ʒvi. of the sulphate; 8 grains being equivalent to an ounce of bark.—Tr."

have carried the dose much higher, but this is seldom necessary. Large doses, indeed, have produced inconvenience, if not danger, giving rise to strong cerebral excitement. A wine or tincture of the sulphate of quinine may be made thus:—Sulphate of quinine, 10 grains; Madeira wine, two pints. The tincture—sulphate of quinine, five grains; alcohol, one ounce.

The cinchonine, while it is much dearer, is not so efficient as the quinine, and, therefore, we shall not dwell on it in this place.

VI. *Veratrine.*—This active substance has been found in the veratrum sabadilla, the colchicum autumnale, and in the common white helebore. It is scarcely soluble in cold water, and requires a thousand times its weight of boiling water for its solution. It is very soluble in ether, and still more so in alcohol. It is insoluble in the alkalis, but soluble in all the vegetable acids which it saturates, forming with them uncrystallizable salts, taking the appearance of gum on evaporation.

*Physiological Action on Animals.*—One or two grains of the acetate of veratrine introduced into the gullet of a dog produces, immediately, abundant salivation, which continues for some time.

"If a small quantity be thrown into any part of the intestinal canal, and the body be opened to observe the effects, the intestine is found to become much indurated, and to relax and contract alternately for a certain time. The part of the mucous membrane which comes in contact with the veratrine is inflamed; the irritation spreads, and vomiting and purging are produced. In a much larger dose the substance induces a very great acceleration of the circulation and of respiration, which is soon followed by tetanus and death." 60.

In a note, in this place, Mr. Haden states, that colchicum, when given in too large a dose, induces always inflammation of the mucous membrane of the bowels. He, also, gives the following fatal case of gout, from an over-dose of tincture of colchicum.

"Mrs. ——, aged forty, after frequently suffering from gout, requested her medical man to give her the colchicum in a very severe fit.

"She took ʒiiss. of a tincture made by infusing ℥iv. of the root in ℥viij. of proof spirit for three days, the mixture being kept at nearly 100° of temperature. This was given in the morning of Dec. 5. In the evening it had produced no effect, except slight qualmishness. Calomel gr. iij. opii gr. i. was ordered at bed-time, and a purging draught for the morning. However, in the night, vomiting and purging commenced, and continued all the next day, in spite of effervescing volatile saline draughts with opium; so that in the evening of the 6th, opii gr. i. camphor gr. iii. were given and repeated in two hours.

"On the 7th, from accident, she was not seen till three *p.m.* when she was found in the collapse preceding death. The gout had previously gradually subsided. It was stated that she became faint at two o'clock *p.m.* and not till then were her friends alarmed. By opium and spirits warmth was reinduced upon the extremities, and a feeling of greater comfort produced; but the pulse never completely recovered, although the sickness was completely subdued; so that at ten *p. m.* she fell into an apoplectic kind of sleep, which terminated in death before morning.

"It is peculiar, in this case, that Mrs.—— was delicate, and some years before had nearly suffered death from incessant vomiting attended by cold extremities; it was relieved by inducing gout on the swelled knee by mustard cataplasms. In the fatal attack the sinapism was applied, with the effect of producing great pain, but without inflammation or heat of skin.

"It should be mentioned also, that this female's mother is exceedingly susceptible of the action of colchicum, in even very small doses. The attendant practitioner begged also to add, that he only prescribed so large a dose as ʒiiss. because the tincture had only been made three days, and the formula directed it should be infused a fortnight." 63.

When we bear in mind that retroceded gout often produces all the phenomena enumerated in the above case, and where no colchicum is given, we really can see no unequivocal proof of the medicine being the cause of death in this case. At the same time we are very far from advocating such huge doses as two drachms and a half of tincture of colchicum under any circumstances.

Veratrine has not been used in the human subject in large doses. A quarter of a grain rapidly induces very abundant alvine evacuations, and if the dose be augmented it occasions more or less violent vomiting. M. Magendie lately gave it in the dose of two grains in the 24 hours, without producing too many alvine evacuations.

VII. *Hydrocyanic Acid.*—This medicine is now so well known as nearly to be forgotten in this country. We shall therefore take no notice of it in this place.

Passing over solanine and delphine, the former an alkali obtained from the berries of deadly nightshade, and the latter a salt from the seeds of stavesacre, we come to—

VIII. GENTIANIN.—For the process of making this, we refer to the work itself, which all operative chemists and apothecaries will, of course, possess. We shall merely glance at the properties of the new remedies. Gentianin is yellow, inodorous, and possesses very strongly the aromatic bitterness of gentian, especially when dissolved in an acid. It appears to have no poisonous quality—and, as far as we can see in Magendie's work, it possesses little, if any, advantage over the root from whence it is obtained by a troublesome process.

IX. *Iodine.*—This is a simple body, discovered in 1813, in the mother waters of soda, as it is obtained from sea-weed. It is solid at the ordinary temperature, in the form of small greyish crystals, having the aspect of plumbago. It is soluble in ether and in spirit of wine. It forms an acid with hydrogen, and another with oxygen. Hydriodic acid may be united to a number of bases, forming neutral salts with them, of which the hydriodate of potash has been that most commonly employed in medicine. M. Coindet, of Geneva, was one of the first who employed it extensively, and, as we said in a former number of this Journal, he has entirely desisted from its internal administration. Externally applied in the form of an ointment, it is one of the most powerful remedies we possess for discussing scrofulous and other glandular tumours.*

X. *Brucine.*—This salt was discovered in the bark of the false angustura bark (brucia anti-dysenterica) by Messrs. Pelletier and Caventou in 1819. It has since been found combined with strychnine in the nux vomica. It is extracted from the bark by a process similar to that by which the strychnine is obtained. It is intensely bitter, and little soluble in water. It presents itself under the form of oblique prisms with parrallelogrammic sides. It unites with acids, forming with them neutral salts. Its action on the animal economy is similar to that of strychnine, but less energetic. It requires four grains of brucine to kill a rabbit. It might be substituted, thinks M. Magendie, for strychnine, with advantage.

We cannot better conclude our notice of this useful little work than in the words of the translator.

"Time alone can pronounce definitively on the advantages and inconveniences of these new remedies; but which ever way it may be, the following pages may be useful by teaching the mode of preparing them without making it necessary to consult general treatises of chemistry or

---

* FORM OF OINTMENT.

℞. Hydriod. Potassæ . . . . ℈ij.

Axungiæ . . . . . . . ℥iss.

Liq. Potass. Caust. . . gtt. iv.

Ft unguentum.

pharmacy, and by giving medical men every facility in submitting them to personal experience which is often after all the only really profitable course."—*Preface* xi.

Since the above analysis was closed, we have learned with regret, though not with surprise, that the zealous and intelligent translator has paid the debt of Nature, while in the Mediterranean with Sir William Curtis, as that gentleman's medical attendant. We saw Mr. Haden a few days before he embarked, and he evidently laboured under aneurism of some of the large vessels in the chest—or some tumour pressing on the lower part of the trachea, or about the bifurcation of the same into the bronchia. His *inspirations* were long and laborious, as if the air was drawn with difficulty through a flattened tube. The breathing was, also, accompanied by a peculiar hissing noise, indicative of the obstructed canal through which it passed. Mr. H. had long laboured under painful sensations in the region of the arteria innominata or subclavian of the right side, and his medical friends had long suspected aneurism in that neighbourhood. Their prognosis was, doubtless, but too soon verified. Mr. Haden always appeared to us to be a man of great zeal, considerable talent, and scrupulously upright character. His attachments, however, of a friendly and medical nature, appeared to us rather romantic. His enthusiastic admiration of every thing French, after spending a few weeks in Paris, rendered his writings, from that period, quite tiresome, and detracted somewhat from the estimate which had been previously formed of his judgment. While we notice this little failing of the head, we are ready to do ample justice to the goodness and integrity of his heart.

---

P. S. Before we dismiss the subject of New Remedies, we may be permitted to say a few words respecting New Pharmacopœias, of which a whole host will have appeared ere these sheets can see the light. The note of preparation, during the last two years, never induced us to form extravagant anticipations of the Official Pharmacopœia of the College, and, therefore, we experienced no poignant disappointment at its appearance. This has evidently not been the case with others. We have long been of opinion, that the conjoint work of *many* heads can never be equal to the offspring of *one* good one. In the conclaves or councils of public bodies there must ever be too much clashing of opinion and difference of sentiment (especially on medical subjects) to prevent the ejection of some good, and the introduction of some evil, into their resolutions. These embarrassments must, consequently, render the compositions emanating from such bodies below the level of public expectation. We shall illustrate this by a naval paradox. If ten of the *fastest* sailing frigates in the royal navy be ordered to proceed with all possible dispatch from Spithead to Madras, *as a squadron*—and if, at the same time, the *slowest* sailing frigate in the navy be ordered to proceed *as a single ship*, she will be at Madras many days, and probably some weeks, before the fast sailing squadron. And why? Because no two ships sail exactly alike, and, therefore, there is such continual backing, filling, trimming, and manœuvring, *in the squadron*, that the single ship, however slow in sailing compared with any *one* of the others, outstrips them all in the long run. How far this simile is applicable to the present subject, let the National Pharmacopœias of France and England bear testimony. In the mean time, while we consider a general standard in the preparation of medicines a very proper thing, we attach far less importance to it than our neighbours. Whether the College execute ill or well their pharmacopœia, the circumstance will neither accelerate nor retard the march of Science. Who, we ask, will prescribe *garlic*, because it has a seat in the pharmacopœia—and reject the *sulphate of quinine*, because it is thought unworthy of a place with the fetid hellebore, madder, and various other sleeping partners of the great firm?

The two National Pharmacopœias of France and England have run into opposite extremes. The former is a chaos, a *rudis indigestaque moles*, of all kinds of unutterable things. The latter has acted with, perhaps, too much caution, both in expulsion and admission. This extreme, however, is infinitely preferable to the other—in fact, we hardly consider it deserving of censure.

While the birth of the New Pharmacopœia has drawn forth a general note of lamentation from our cotemporaries, we confess that it has had quite a contrary effect upon us. We could not but be amused at the tiptoe expectation evinced among the host of critics, commentators, and counsellors—nor could we resist the temptation to laugh at their long blank countenances, when they saw their criticisms, comments, and friendly advice thrown away! One of two things is certain: either the voice of the critics never penetrated the sanctum sanctorum of the College—or, if it did, it was there stricken as mute as the marble busts of Harvey and Sydenham.

The additions and subtractions in the new edition are far from numerous. Into the Materia Medica six new articles have been admitted—cubebs, elecampane root, the garden lettuce, seeds and leaves of strammonium, oil of croton, and rhatany root. The *seeds*, also, of conium, colchicum, and digitalis, are now admitted. Two drugs have disappeared from this department—aloe vulgaris and vinum album Hispanicum. Some alterations and improvements have taken place in the department "*Preparata et Composita,*" but only one single formula has been thrown out, the oxydum antimonii. The other changes are few in number, and of no very peculiar interest. But, as critical commentaries are issuing from the press in great numbers, some of which it will be our duty to notice hereafter, we shall dwell no longer on the subject. We think the College has pruned with a rather too sparing hand, and that they might have admitted a few formulæ, now extemporaneously employed, which might have advantageously supplied the places of those which they could have thrown out. But, as we said before, they have acted on the side of caution, and, consequently, of safety. We cannot, therefore, join in the hue and cry against the New Pharmacopœia.

